# Ultrathin Microlens Arrays for Dynamic Beam Shaping Based on 3D Lithography

**DOI:** 10.3390/mi17020250

**Published:** 2026-02-16

**Authors:** Ruiqi Cheng, Yue Zhang, Shuo Chen, Yu Shu, Hao Cao, Chengqun Gui

**Affiliations:** 1 School of Integrated Circuits, Wuhan University, Wuhan 430072, China; 2 Hubei Key Laboratory of Electronic Manufacturing and Packaging Integration, Wuhan University, Wuhan 430072, China

**Keywords:** 3D lithography, low-aspect-ratio, beam shaping, dynamic tuning

## Abstract

Conventional microlens arrays (MLAs) are often constrained by their static focal properties, which limit post-fabrication adaptability in dynamic optical systems. To address this, we demonstrate a tunable beam shaper capable of real-time spot-size modulation by introducing an adjustable axial displacement between a primary lens and an MLA. A critical advancement of this work is the fabrication of ultra-thin MLAs featuring an exceptionally low aspect ratio (1:187.5) and continuous surface profiles. Through optimizing 3D lithography and ion beam etching (IBE) workflows, we achieved an optical-grade surface finish with a roughness (*Sa*) of 3 nm. This high-fidelity, low-profile component enables efficient beam homogenization with reconfigurable working distances and spot dimensions. The proposed architecture provides a versatile and robust solution for advanced laser material processing, bridging the gap between static beam shaping and dynamic laser delivery.

## 1. Introduction

While laser technology has emerged as the primary catalyst for modern precision manufacturing, the intrinsic non-uniformity of fundamental Gaussian beams—characterized by a high-intensity core and rapid peripheral decay—presents a critical physical bottleneck that constrains the upper limits of processing efficiency. This energy distribution non-homogeneity can lead to severe consequences in high-end manufacturing and precision medicine [1,2,3,4,5]. The excessive peak power density at the Gaussian center often triggers dynamic instability in the melt pool keyhole, where fluctuations in recoil pressure from metal vapor induce violent spattering and porosity defects, ultimately compromising the integrity of the welded joint [6]. Similarly, in laser additive manufacturing (AM) and cladding, the spatial distribution of thermal input dictates the evolution of the solidification microstructure. Recent studies show that traditional Gaussian beams typically generate deep, narrow melt pools that favor coarse grain growth along the heat flow direction; conversely, a reshaped beam facilitates a more uniform thermal field, promoting a transition to fine equiaxed grains and significantly enhancing the mechanical properties of the material [7,8,9,10]. Furthermore, in medically sensitive scenarios such as ophthalmological surgery, even minor local energy fluctuations can exceed safe thermal damage thresholds, causing irreversible biological tissue damage [11].

Given these limitations, transforming a Gaussian beam into a top hat profile with uniform irradiance has become an essential pathway for advancing process quality. While diverse beam-shaping architectures, such as diffractive optical elements (DOEs), metasurfaces, and metalenses, have been proposed to address this, microlens arrays (MLAs) have established a dominant role in industrial beam homogenization due to their superior engineering robustness [12,13,14,15,16,17]. The operational mechanism of an MLA is based on the “beam integration” principle: the system spatially discretizes the incident beam into a multitude of sub-beams via microlens units, which are subsequently superimposed incoherently at the target focal plane by a condenser lens. This process statistically “smooths” the random intensity fluctuations of the original beam, synthesizing a uniform spot with a high degree of flatness [18]. Compared to diffractive optical elements (DOEs), which are highly sensitive to environmental conditions, MLAs exhibit exceptional field adaptability. Due to the integration principle, these systems possess high immunity to wavefront aberrations, mode hopping, and positional jitter, maintaining stable output even in complex, high-vibration workshop environments [19,20,21]. The evolution of optical beam shaping has been significantly marked by the emergence of metasurface platforms, which offer sophisticated control over light-matter interactions within sub-wavelength footprints. Recent advances in metasurfaces have realized non-mechanical multifunctionality via phase-change materials, self-accelerating beam generation for on-chip integration, and high-fidelity amplitude modulation through 3D nanoprinting [22,23,24]. While these planar architectures excel in compact multifunctionality and intricate wavefront tailoring, their industrial utility is often curtailed by the resonant nature of their constituent elements, which impose bandwidth constraints and lower laser-damage thresholds. In contrast, as refractive components with smooth surfaces, MLAs circumvent dispersion and exhibit exceptional damage thresholds, making them essential for kilowatt-level operations. By prioritizing broadband transparency and thermal stability over extreme miniaturization, our strategy ensures the robust power-handling and longevity required for intensive industrial processing. This approach provides a resilient pathway for beam homogenization under extreme irradiance, especially where material stability is the primary operational constraint [25,26,27]. The efficacy of high-precision MLAs is fundamentally contingent upon the sophistication of advanced fabrication methodologies. Although techniques such as femtosecond laser micromachining and electron-beam lithography (EBL) facilitate intricate geometries, suppressing spherical aberrations for peak homogenization typically necessitates ‘shallower’ geometries with reduced aspect ratios—a constraint that places stringent demands on the *Z*-axis resolution of the patterning system. Given that inherent fabrication artifacts, such as proximity effects, can inadvertently distort the intended topography, robust optimization strategies are imperative for safeguarding structural fidelity. Following fabrication, stringent surface metrology becomes a prerequisite to ensure the final refractive element faithfully replicates the prescribed theoretical design [28,29,30,31].

However, the fixed magnification of conventional MLAs often limits system flexibility; thus, developing a tunable architecture capable of dynamic spot modulation is essential for multi-functional laser processing. To address the inherent rigidity of conventional beam homogenizers, this paper introduces a compact, reconfigurable architecture capable of continuous spot-size modulation. By engineering the inter-element axial separation between a primary objective and a microlens array (MLA), we demonstrate a method to decouple output irradiance dimensions from the physical constraints of static optics. The realization of this system hinges on a refined fabrication pipeline—utilizing 3D laser lithography in conjunction with optimized ion beam etching—to produce shallow-profile MLAs with near-atomic surface quality. We show that achieving a surface roughness of 3 nm is critical for operating within the smooth-surface regime, thereby minimizing detrimental scattering. This approach provides a versatile and robust framework for tailoring laser beam profiles, offering significant advantages for high-precision manufacturing and adaptive optical systems.

## 2. Design and Calculate

### 2.1. Limitations of Conventional Microlens Beam Homogenizers

**Figure 1:**
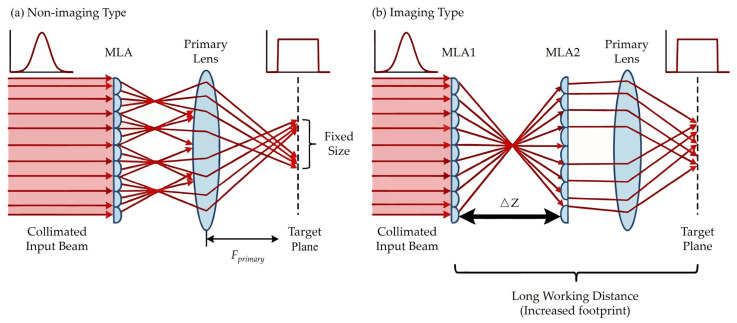
Schematic diagram of common microlens beam homogenizers: (**a**) non-imaging configuration with fixed parameters; (**b**) imaging configuration featuring a double-MLA structure for extended working distances.

Conventional MLA-based beam homogenizers are typically categorized into imaging and non-imaging configurations, as illustrated in Figure 1a,b. Both architectures fundamentally rely on a shared mechanism: the incident beam is partitioned into sub-apertures by the microlens array, which are subsequently superimposed by a condenser lens at the target plane to produce a uniform irradiance distribution.

Geometric optical analysis indicates that for non-imaging homogenizers, the final spot dimensions are strictly governed by the aperture sizes of both the sub-lens and the condenser lens. This rigidity implies that once the device is fabricated, its working distance and output footprint remain static. Consequently, varying application requirements necessitate bespoke design and fabrication cycles, significantly exacerbating both computational overhead and manufacturing costs. While imaging homogenizers offer spot-size adjustment via the inter-lens spacing of the MLA pair, their requisite three-element architecture substantially increases the system’s total optical path length, thereby limiting their utility in compact or space-constrained environments.

### 2.2. Design of a Tunable Microlens Beam Shaper

To address the need for versatility across different operational scenarios, we propose a compact beam-shaping scheme that offers dynamic control over the output spot size. While imaging-type systems achieve tunability via the relative displacement of twin MLAs, standard non-imaging designs are typically limited by fixed divergence angles once the lens parameters are set. To introduce a degree of freedom into the non-imaging framework, this design strategically reorders the primary lens and the MLA. This modification allows the output spot size to be precisely governed by the distance between the two elements. Such a configuration maintains a minimal component count while providing the necessary adaptability for varying working distances and spot specifications. This configuration essentially alters the incident wavefront curvature on the MLA, thereby shifting the far-field distribution as a function of the inter-element distance.

**Figure 2:**
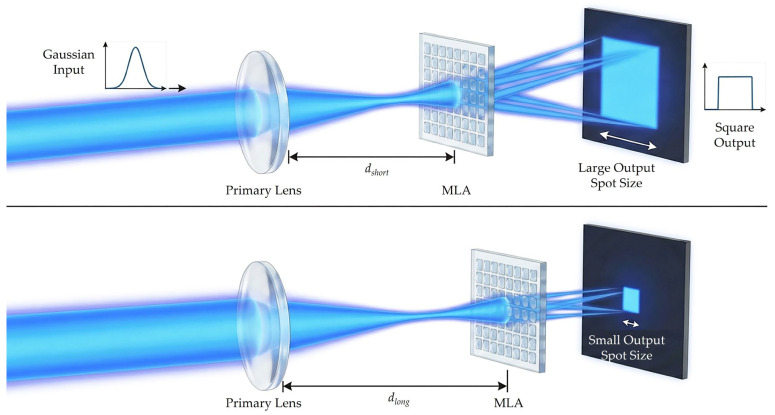
Schematic illustration showing the principle of tunable output spot size achieved by varying the axial distance *d* between the primary lens and the MLA.

In the proposed tunable MLA-based beam shaper, as shown in Figure 2, the incident light first undergoes wavefront flattening via the primary lens. This pre-collimation expands the beam footprint, ensuring that a greater number of MLA sub-lenses are illuminated to improve homogenization. Given a fixed target plane (situated at the focal length of the primary lens) and a constant divergence angle from the MLA, the resulting spot size is sensitive to the axial positioning of the components. Specifically, an increase in the axial separation between the primary lens and the MLA leads to a proportional reduction in the output spot dimensions. By leveraging this axial displacement as a tunable degree of freedom, the system achieves variable magnification, functioning as a high-performance projection homogenizer with a dynamically adjustable output.

**Figure 3:**
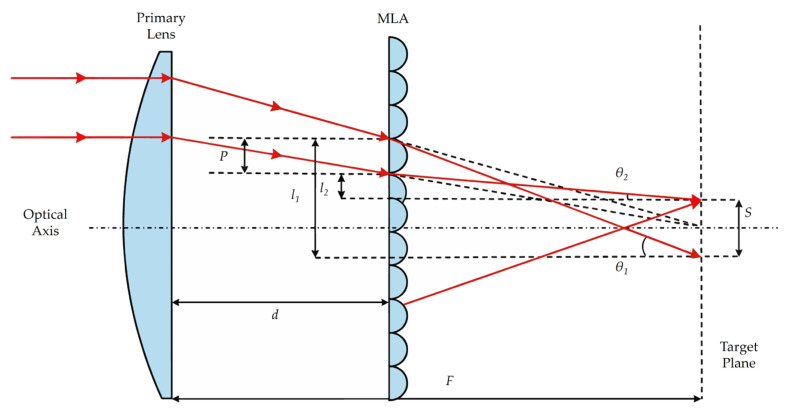
Schematic diagram of the tunable beam shaping configuration, illustrating the geometric parameters that define the optical path.

The optical layout and the definition of various parameters are illustrated in Figure 3. With a Gaussian beam as the input and a rectangular flat-top profile as the target output, each lenslet within the MLA is defined by a rectangular pitch *P* and a radius of curvature *R*. We performed a marginal ray-tracing analysis for the boundary of the individual MLA lenslet. For microlens arrays with a low-aspect-ratio, where the lenslet dimensions are significantly smaller than the radius of curvature, the paraxial approximation is valid. To rigorously assess the validity of this approximation, we performed a third-order Taylor expansion on the trigonometric functions, thereby incorporating the relevant error terms $\frac{\theta^{3}}{6}$. Consequently, the relative error can be quantified by $\varepsilon =\frac{\left|\Delta \theta \right|}{\theta }\approx \frac{\theta^{2}}{6}$. Consequently, the relationship between the incident and emergent ray angles through the MLA is given by the following equation:

(1) $$\theta_{in}=sin{\;\theta }_{in}=tan\;\theta_{in},$$

(2) $$\theta_{out}=sin{\;\theta }_{out}=tan\;\theta_{out},$$

Let *h* and *n* represent the incidence height and the refractive index of the constituent lenslets, respectively. Incorporating Snell’s law within the established geometric framework, we derive the following relationship:

(3) $$sin{\;\theta }_{out}=n\cdot sin{\;\theta }_{in},$$

(4) $$\theta_{in}=\frac{h}{R},$$

(5) $$\theta =\theta_{out}-\theta_{in},$$

The combination of Equations (1)–(5) yields:

(6) $$tan\;\theta_{1}=\frac{h_{1}}{R}(n-1),$$

(7) $$tan{\;\theta }_{2}=\frac{h_{2}}{R}(n-1),$$

Geometric considerations reveal that:

(8) $$l_{1}=tan\;\theta_{1}\cdot (F-d),$$

(9) $$l_{2}=tan{\;\theta }_{2}\cdot (F-d),$$

(10) $$P=l_{1}-l_{2}-S=h_{1}-h_{2},$$

By consolidating the relations defined in Equations (6)–(10), we arrive at the following characterization for the spot size *S*:

(11) $$S=f\left(P,F,R,n,d\right)=P\cdot [(n-1)\cdot \frac{F-d}{R}-1],$$

Evidently, the dimensions of the final output spot are governed by the lenslet aperture, the focal lengths of both the MLA and the primary lens, and the tunable parameter *d*. This implies that once the hardware specifications are fixed, the spot size can be dynamically tailored by modulating *d*. Such versatility allows the system to meet diverse working distance requirements while precluding the need for the costly re-fabrication of homogenization components.

To clarify the formation of the square profile, the propagation of a coherent laser field through the system is modeled using the principles of Fourier optics. The process begins with a collimated incident laser field, denoted as $U_{\mathrm{in}}(x,y)$. Upon passing through the Fourier Transform (FT) lens with focal length *F*, the wavefront undergoes a quadratic phase transformation:

(12) $$t_{FT}(x,y)=\exp[-j\frac{k}{2F}(x^{2}+y^{2})]$$

where $k=\frac{2\pi }{\lambda }$ represents the wavenumber. This modulation imparts the necessary convergence to the field, mapping the subsequent angular deviations of the microlens array into spatial coordinates at the focal plane. The MLA serves as the primary homogenization element. Its complex transmittance, $t_{MLA}(x,y)$, is defined by the periodic arrangement of individual square lenslets. Each lenslet within the array provides both spatial truncation and a localized phase shift:

(13) $$t_{sub}(x,y)=rect(\frac{x}{P},\frac{y}{P})\cdot \exp[-j\frac{k}{2f_{mla}}(x^{2}+y^{2})]$$

where *P* represents the pitch of the lenslet, $f_{mla}$ represents its individual focal length. The total transmittance of the MLA is the convolution of the single-lenslet function with a Dirac comb, representing the periodic lattice:

(14) $$t_{MLA}(x,y)=t_{sub}(x,y)⊗\sum_{m,n}\delta (x-mP,y-nP)$$

The complex amplitude at the focal plane, $U_{f}(u,v)$, is proportional to the Fourier transform of the field exiting the MLA. Utilizing the convolution theorem, the resulting irradiance $I(u,v)={\left|U_{f}\right|}^{2}$ is expressed as the product of two distinct physical distributions:

(15) $$I(u,v)\propto {\left|F\left\{t_{sub}(x,y)\right\}\right|}^{2}\cdot {\left|F\left\{\sum_{m,n}\delta (x-mP,y-nP)\right\}\right|}^{2}$$

The first term, ${\left|F\left\{t_{sub}(x,y)\right\}\right|}^{2}$, determines the intensity envelope. Because the Fourier transform of a rect function is a sinc function (modulated by the lenslet’s quadratic phase), it creates a square-shaped region of uniform energy.

The second term, ${\left|F\left\{\sum_{m,n}\delta (x-mP,y-nP)\right\}\right|}^{2}$, represents the interference pattern. The Fourier transform of a periodic Dirac comb is another Dirac comb in the frequency domain.

## 3. Results and Discussion

### 3.1. Numerical Simulation and Feasibility Verification

**Figure 4:**
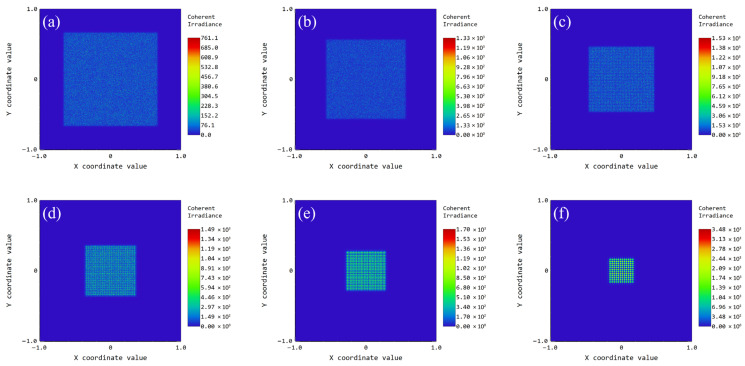
Simulated irradiance distributions of the rectangular spot at various values of the tunable parameter *d* (both x and y axis units are in 1 mm): (**a**) *d* = 15 mm; (**b**) *d* = 25 mm; (**c**) *d* = 35 mm; (**d**) *d* = 45 mm; (**e**) *d* = 55 mm; (**f**) *d* = 65 mm.

To validate the theoretical framework, we implemented a non-sequential ray-tracing model in ZEMAX 2019.4 to quantify the sensitivity of the spot dimensions to the displacement *d*. The parameters for the incident beam and the MLA are summarized in Table 1. By modulating the axial separation between the primary lens and the MLA, we observe that the spot size at the focal plane evolves in response to the tunable variable *d*. Crucially, the total track length of the system remains fixed during this process, further substantiating the validity of the theoretical framework. The quantitative relationship and the captured intensity profiles are presented in Table 1 and Figure 4a–f. The light source is configured as a 532 nm Gaussian beam with a radius of 2 mm. The refractive index of the HPFS 7980 fused silica is approximately 1.46 at the operating wavelength of 532 nm. To maintain a relative error of less than 1% when applying the paraxial approximation to an MLA, the maximum aperture angle must be restricted to under 0.245 rad, which corresponds to an aspect ratio of less than 1:16. The MLA developed in this study possesses an aspect ratio of 1:187.5; consequently, the resulting approximation error is significantly suppressed and remains far below the 1% threshold.

A detailed analysis of the results presented in Table 1 and Figure 4a–f confirms that the beam-shaping performance remains robust across the entire adjustment range. As the tunable parameter *d* increases, the spot size scales predictably while maintaining a high degree of irradiance uniformity, indicating that the paraxial approximation used in our theoretical model is highly accurate for this shallow-profile MLA configuration. These findings provide a solid computational benchmark, ensuring that the fabricated device will perform reliably under real-world operational constraints.

### 3.2. Fabrication of Low-Aspect-Ratio Microlens Arrays

Based on the optimized parameters yielded by our simulations, we proceed to the fabrication of the optical components.

#### 3.2.1. Fabrication Process

**Figure 5:**
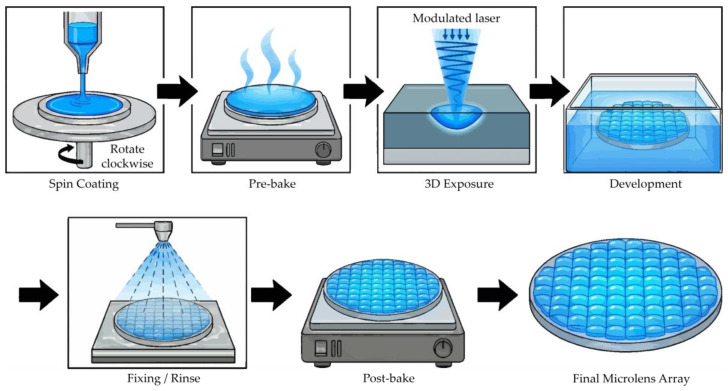
Schematic illustration of the single-step 3D grayscale lithography process for defining the microlens array morphology on photoresist.

Realizing architectures with extreme aspect ratios typically entails intricate and time-intensive fabrication processes. The microlens arrays (MLAs), characterized by continuous surface profiles and exceptionally low-aspect-ratio, were fabricated via a single-step 3D lithographic strategy [32,33]. The underlying mechanism is schematically illustrated in Figure 5. Initially, the photoresist was spin-coated onto a cleaned glass substrate, followed by a pre-bake on a leveled hotplate to stabilize the film. Following the pre-bake process, the samples were allowed to reach thermal equilibrium at ambient temperature. This stabilization step was essential to ensure a consistent photoresist response and prevent thermal fluctuations prior to initiating the 3D lithographic exposure.

**Figure 6:**
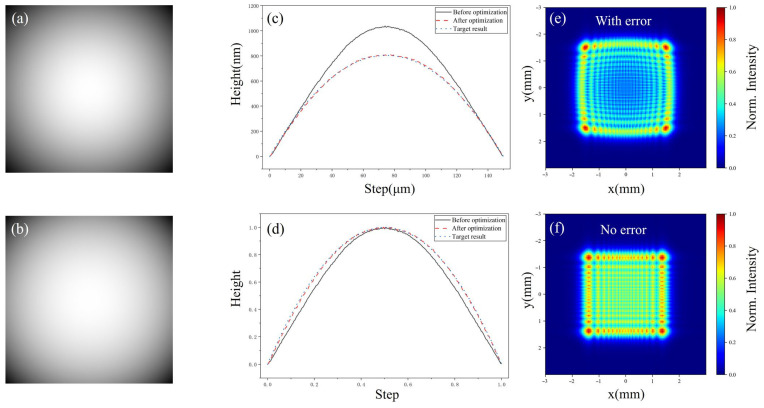
Grayscale map optimization, the resulting surface profiles, and physical optics transport simulation results by Python 3.12: (**a**) grayscale map of the MLA unit cell before optimization; (**b**) grayscale map after optimization; (**c**) comparison of the lithographic sag profiles and (**d**) normalized sag profiles before and after optimization; (**e**) simulated spot results with morphological errors; (**f**) simulated spot results without morphological errors.

The structural fidelity of 3D micro-nanostructures is frequently compromised by the depth-dependent attenuation of laser intensity within the photoresist, a phenomenon compounded by the non-linear response of the resist to exposure dosage. These factors typically induce morphological warping that inevitably undermines the optical performance of the resulting devices. A 10% profile error induces significant pincushion distortion and outward protrusion of the spot, alongside a decrease in central uniformity (as shown in Figure 6e,f). To counteract these discrepancies, we implemented a 3D OPC strategy based on the SinCUT algorithm [34]. This method computationally refines the grayscale maps to compensate for proximity effects and resist response, ensuring the final morphology closely aligns with the theoretical design. Figure 6a,b displays the grayscale maps of the MLA unit cell before and after optimization, respectively. It can be observed that the optimized map incorporates compensated gray levels to mitigate the nonlinear response of the photoresist. The cross-sectional profiles of the fabricated MLA are characterized to evaluate the pattern transfer accuracy. As shown in Figure 6c, the lithographic sag profile obtained from the optimized grayscale map exhibits a closer agreement with the design target compared to the non-optimized one. To further investigate the shape fidelity, the normalized sag profiles are plotted in Figure 6d. The result reveals that the optimized profile significantly reduces the deviation at the lens edge, demonstrating that the proposed optimization method effectively enhances the surface profile accuracy during the 3D lithography process. Following a post-exposure bake (PEB), development, and fixing, the 3D structures were accurately patterned in the photoresist layer.

Subsequently, these resist patterns were transferred into the Corning C79-80 glass substrate via Ion Beam Etching (IBE). To account for the etch selectivity—where the ratio of the substrate’s etch depth to that of the photoresist is approximately 1:0.78—the 3D OPC-optimized grayscale maps were pre-scaled accordingly. This precise scaling ensured the final glass structures replicated the intended specifications.

#### 3.2.2. Fabrication Parameters

Corning 7980 high-purity fused silica (2.5 mm thick) was selected as the substrate due to its exceptional refractive index homogeneity and broad transmission spectrum. To eliminate sub-visible particulates, the substrates underwent a rigorous two-stage cleaning protocol. Initially, 50 kHz ultrasonic agitation was utilized to remove particles on the surface. This was followed by oxygen plasma treatment, which served to eliminate residual organic contaminants and activate the surface, ensuring robust interfacial adhesion for the subsequent photoresist coating. Prior to coating, the photoresist was degassed via centrifugation at 3000 rpm (corresponding to a relative centrifugal force of 1005× *g*) for 8 min at 18 °C to ensure a bubble-free film.

Given the target structure depth of 800 nm and the established etch selectivity, the required relief depth in the photoresist was calculated to be 624 nm. Consequently, S1818 thin-film photoresist was utilized. To account for the exposure threshold and potential resistance to “sinking” during development, the target spin-coated thickness was set to approximately 1.6 μm. A two-step spin-coating procedure was executed: an initial spread at 500 rpm (100 rpm/s acceleration) for 15 s, followed by a ramp to 4000 rpm (1000 rpm/s acceleration) for 60 s to achieve the desired film uniformity. The average thickness of the fabricated photoresist layer was recorded at 1653 nm. Based on profilometry measurements, the within-wafer thickness variation was strictly maintained within ±2%.

Exposure was performed using a 4PICO PM-250 laser direct writing system operating at 365 nm. The exposure parameters were finely tuned with a dose of 100 mJ/cm^2^, a 601 nm spot size, a 300 nm step resolution, and a scanning speed of 100 mm/s. Following exposure, the patterns were developed using a standard tetramethylammonium hydroxide (TMAH) solution. The final pattern transfer into the glass was executed via Ion Beam Etching (IBE) using a MIBE-200C system. The process employed Argon ion bombardment at a grazing angle of 45°, maintaining a stable etching rate of 18.8 nm/min. Prior to the process, the chamber was evacuated to a base pressure of 5 × 10^−4^ Pa to ensure a high-vacuum environment. The etching was performed under the following optimized conditions: a cathode current of 10.6 A, an anode voltage of 40 V, a screen voltage of 400 V, and a neutralizer current of 8 A.

### 3.3. Morphological Characterization and Structural Analysis

**Figure 7:**
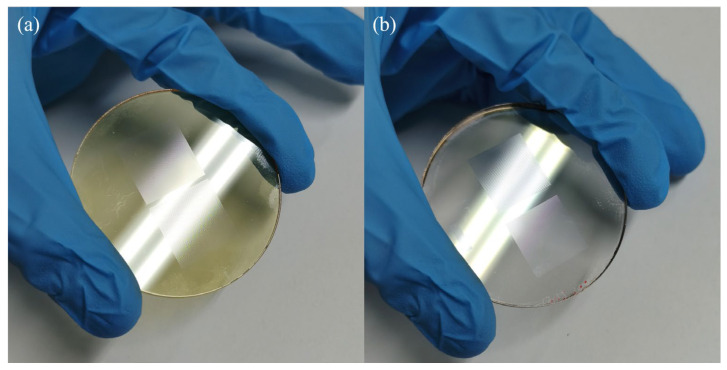
Characterization of the fabricated microlens arrays (MLAs): (**a**) low-aspect-ratio MLA pattern formed on photoresist via 3D lithography; (**b**) final low-aspect-ratio MLA transferred onto the glass substrate surface using ion beam etching (IBE).

Figure 7a,b presents the final fabricated microlens arrays (MLAs) with an ultra-low aspect ratio, demonstrating high-fidelity surface morphology achieved through the optimized 3D lithography and subsequent etching processes.

**Figure 8:**
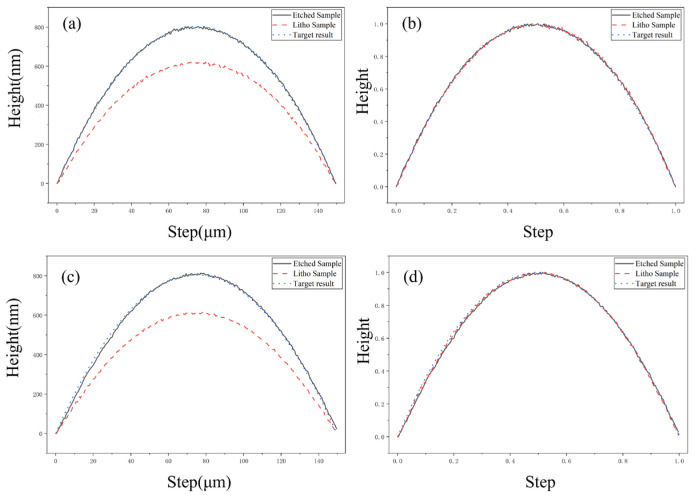
Measured surface profiles of the samples before and after etching: (**a**) comparison of central profiles; (**b**) normalized central profiles; (**c**) comparison of edge profiles; (**d**) normalized edge profiles.

The etching fidelity and isotropy were further evaluated by comparing the central and edge contours of the etched samples against their photolithographic templates Figure 8a,d. The high degree of overlap in the normalized profiles confirms a nearly uniform etch rate across all orientations, characteristic of highly isotropic etching behavior. This synergy between 3D grayscale lithography and Ion Beam Etching (IBE) offers an effective pathway for the precision manufacturing of low-aspect-ratio microlens arrays (MLAs).

**Figure 9:**
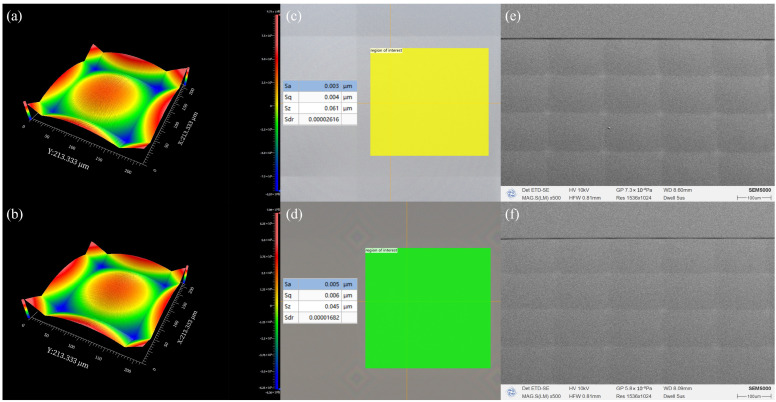
Surface characterization and morphological comparison of the fabricated photoresist (PR) templates and quartz micro-structures. 3D surface topographies obtained by white-light interferometry for (**a**) the etched quartz structure and (**b**) the PR template. Surface roughness evaluation and high-frequency residuals analyzed via confocal microscopy for (**c**) the etched quartz structure and (**d**) the PR template. Scanning electron microscopy (SEM) images captured at a 45° tilt angle of (**e**) the etched quartz structure and (**f**) the PR template.

The 3D topographies of the quartz and photoresist (PR) structures were characterized via white-light interferometry, as illustrated in Figure 9a,b, respectively. Detailed surface roughness was assessed using a Keyence VK-X 3000 confocal microscope. To isolate the high-frequency residuals, the measured height data within a 90% effective lenslet area were processed by subtracting the fitted spherical form. Following a filtration process compliant with the ISO 25178 standard [35], the arithmetical mean height (*Sa*) and root mean square height (*Sq*) were derived. As evidenced by Figure 9c,d, the quartz structure achieved an *Sa* of 3 nm and an *Sq* of 4 nm, while the PR template exhibited an *Sa* of 5 nm and an *Sq* of 6 nm. These metrics underscore the superior surface quality attainable through both 3D lithography and IBE under the optimized process parameters. Finally, SEM micrographs of the quartz and PR samples are presented in Figure 9e,f.

These results indicate that the surface roughness remains well below the Rayleigh criterion threshold, ensuring that the MLA operates within the smooth-surface regime with negligible diffuse scattering. The exceptionally low *Sa* confirms the effectiveness of the optimized development parameters and laser direct writing process in suppressing periodic scanning artifacts and stochastic noise. Collectively, the high-fidelity surface finish validates the reliability of this fabrication workflow, ensuring its suitability for high-performance applications such as high-contrast beam shaping and homogenization.

### 3.4. Beam Shaping Performance Characterization

**Figure 10:**
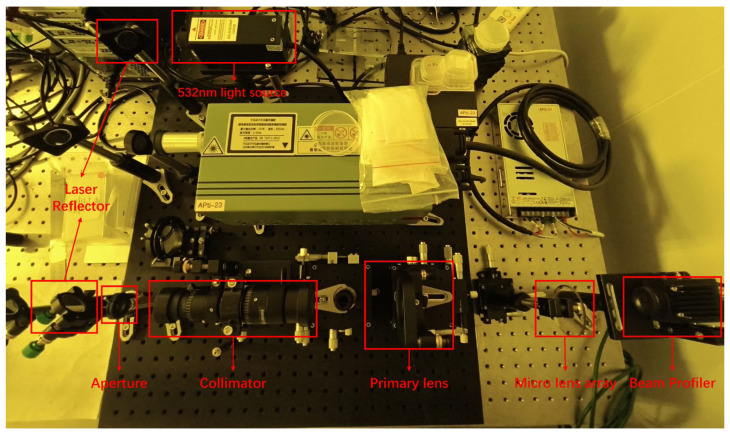
Experimental setup for the characterization of the microlens array.

To validate the theoretical derivations and numerical simulations, and to verify the dynamic spot-sizing functionality of the fabricated microlens array (MLA), an optical characterization platform was constructed as illustrated in Figure 10. A 532 nm Gaussian beam, emitted from a solid-state laser (MGL-III-532), was directed through a series of folding mirrors into a collimator. The collimated beam was then processed by a focusing lens and the MLA, with the resulting shaped profile captured at the focal plane using a beam profiler (SPIRICON BGP-USB3-SP300-OJ).

**Figure 11:**
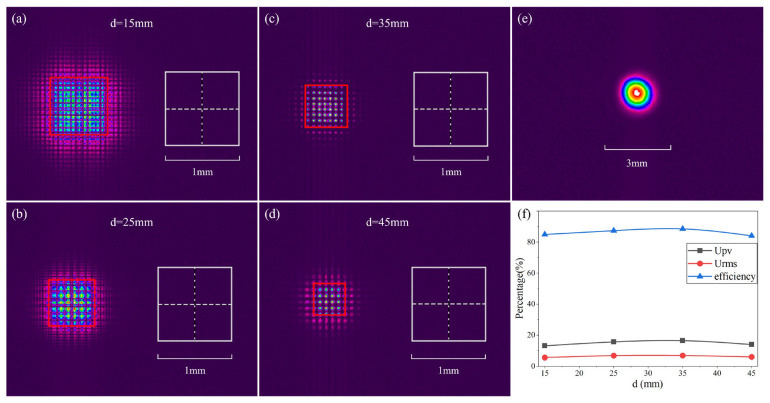
Experimental setup and characterization of the beam-shaping performance of the fabricated microlens array. (**a**–**d**) Morphological evolution of the shaped focal spots captured at varying displacement parameters *d* = 15, 25, 35, and 45 mm, respectively, where the red solid boxes indicate the overall extent; (**e**) intensity profile of the initial incident Gaussian beam provided for comparison; (**f**) quantitative evaluation of the beam-shaping performance.

Figure 11a–d illustrates the morphological evolution of the shaped focal spots captured by the beam profiler across a range of displacement parameters *d* (from 15 mm to 45 mm). When contrasted with the original incident Gaussian profile shown in Figure 11e, these experimental results provide clear evidence of the MLA’s robust beam-shaping performance.

Regarding the beam-shaping performance, the measured profile exhibits a well-defined square geometry, characterized by an internal interference lattice modulated by a square diffraction envelope. This grid-like square distribution demonstrates a high degree of fidelity to the ZEMAX simulation model. As indicated by the 1 mm × 1 mm scale bar, the shaped intensity distribution effectively breaks the rotational symmetry of the incident Gaussian beam, achieving a successful energy redistribution into the target square region.

In terms of the system’s dynamic tunability, experimental data indicate that the parameter *d* exerts a significant modulating effect on the final spot size. As *d* is incrementally increased from 15 mm to 45 mm, the corresponding contraction in the square spot envelope exhibits a downward trend that aligns precisely with the ZEMAX simulation model.

Without altering the overall working distance or modifying the physical dimensions of any optical components, the output spot size can be flexibly scaled solely by adjusting the internal parameter *d*. This ‘non-invasive’ tuning characteristic exhibits a high degree of consistency with the simulation results.

The raw focal spots are subject to significant high-frequency intensity modulation and discrete local extrema caused by interference. Given that these high-frequency oscillations—essentially coherent noise—heavily obscure the macroscopic envelope of the light field, uniformity metrics extracted directly from the raw data often lack a reliable reference value due to statistical bias. Accordingly, we implemented a Gaussian spatial smoothing filter to decouple the underlying macro-envelope from the high-frequency speckle noise. This ensures that the shaping performance of the MLA is accurately captured without being obscured by the stochastic interference fluctuations inherent in coherent systems. The resulting uniformity, root-mean-square (RMS) uniformity, and efficiency (the energy fraction within a fixed region of interest corresponding to 90% of the theoretical spot size), calculated based on these processed energy envelopes, are presented in Figure 11f.

## 4. Conclusions

This research establishes a compact beam-shaping architecture that overcomes the rigidity of conventional homogenizers by introducing a tunable degree of freedom (*d*) to adjust output spot dimensions dynamically. Unlike traditional designs, our approach strategically reorders the optical elements to provide versatile magnification while maintaining a fixed total track length, while ensuring the beam integrity remains substantially uncompromised. A significant contribution of this work lies in the precision manufacturing of ultra-low-aspect-ratio (the height-to-width ratio is 1:187.5) MLAs with high-fidelity continuous surfaces. While 3D OPC was used as a supporting tool to compensate for resist nonlinearity, the core innovation is demonstrated by achieving an arithmetic mean roughness (*Sa*) of 3 nm, which ensures operation in the smooth-surface regime with negligible scattering. This high-fidelity surface finish validates the robustness of our fabrication methodology for shallow-profile optics. However, the strictly periodic arrangement of the MLA sub-units inevitably induces coherent interference effects. The resulting spot morphology—specifically the formation of a discrete lattice of interference peaks—aligns with our earlier simulation assumptions and experimental results, which modeled the input as a highly coherent Gaussian source, thereby confirming the predictive reliability of the established physical optics framework. Future efforts will address this limitation by introducing stochasticity into the array, such as randomizing sub-lens dimensions or heights, to decorrelate sub-beam phases and suppress interference artifacts.

## Figures and Tables

**Table 1:** Main design parameters of the optical system.

Component	Parameter	Value	Description
Primary Lens	Material	HPFS 7980	Type of glass
	Curvature Radius	38.63 mm	Spherical surface radius
	Clear Aperture	1 inch	Effective diameter of the lens
MLA	Material	HPFS 7980	Type of glass
	Curvature Radius	3.5 mm	Radius of each individual lenslet
	Lenslet Pitch	150 μm	Distance between adjacent centers
	Array Configuration	50 × 50	Total number of units
	Total Area	7.5 mm × 7.5 mm	Calculated as 50 × 150 μm

## Data Availability

The data presented in this study are available within the article.

## Abbreviations

The following abbreviations are used in this manuscript:

IBE	Ion beam etching
LSCM	Laser scanning confocal microscopy
MLA	Microlens array
SEM	Scanning electron microscopy
